# Volume rendered 3D OCTA assessment of macular ischemia in patients with type 1 diabetes and without diabetic retinopathy

**DOI:** 10.1038/s41598-021-99297-7

**Published:** 2021-10-05

**Authors:** Enrico Borrelli, Domenico Grosso, Mariacristina Parravano, Eliana Costanzo, Maria Brambati, Chiara Viganò, Riccardo Sacconi, Lea Querques, Adelaide Pina, Daniele De Geronimo, Francesco Bandello, Giuseppe Querques

**Affiliations:** 1 Department of Ophthalmology, University Vita-Salute, IRCCS Ospedale San Raffaele, Via Olgettina 60, Milan, Italy; 2 IRCCS - Fondazione Bietti, Rome, Italy

**Keywords:** Biomarkers, Biomarkers

## Abstract

The aim of this study was to measure macular perfusion in patients with type 1 diabetes and no signs of diabetic retinopathy (DR) using volume rendered three-dimensional (3D) optical coherence tomography angiography (OCTA). We collected data from 35 patients with diabetes and no DR who had OCTA obtained. An additional control group of 35 eyes from 35 healthy subjects was included for comparison. OCTA volume data were processed with a previously presented algorithm in order to obtain the 3D vascular volume and 3D perfusion density. In order to weigh the contribution of different plexuses’ impairment to volume rendered vascular perfusion, OCTA *en face* images were binarized in order to obtain two-dimensional (2D) perfusion density metrics. Mean ± SD age was 27.2 ± 10.2 years [range 19–64 years] in the diabetic group and 31.0 ± 11.4 years [range 19–61 years] in the control group (p = 0.145). The 3D vascular volume was 0.27 ± 0.05 mm^3^ in the diabetic group and 0.29 ± 0.04 mm^3^ in the control group (p = 0.020). The 3D perfusion density was 9.3 ± 1.6% and 10.3 ± 1.6% in diabetic patients and controls, respectively (p = 0.005). Using a 2D visualization, the perfusion density was lower in diabetic patients, but only at the deep vascular complex (DVC) level (38.9 ± 3.7% in diabetes and 41.0 ± 3.1% in controls, p = 0.001), while no differences were detected at the superficial capillary plexus (SCP) level (34.4 ± 3.1% and 34.3 ± 3.8% in the diabetic and healthy subjects, respectively, p = 0.899). In conclusion, eyes without signs of DR of patients with diabetes have a reduced volume rendered macular perfusion compared to control healthy eyes.

## Introduction

The macula is a highly specialized neuronal tissue with an extremely high energy demand. The term diabetic macular ischemia refers to the enlargement of the foveal avascular zone and presence of macular regions of capillary nonperfusion that may occur in diabetic eyes^1^. Diabetic macular ischemia seems to be secondary to selective loss of pericytes and thickening of the basement membrane as a consequence of exposure of these structures to raised blood glucose levels^2^. This represents a frequent complication in diabetes as it occurs in approximately 41% of patients with diabetic retinopathy (DR)^1^.

Importantly, macular ischemia seems to occur early in diabetes as previous studies have demonstrated that this complication may also affect diabetic patients without DR^3–8^. In these patients, dye-based angiography of the retinal vasculature is not routinely performed, since this technique requires intravenous administration of fluorescein dye and performing this exam is not essential in the setting of diabetes and no signs of DR as it does not provide meaningful information at this specific stage. However, the introduction of optical coherence tomography angiography (OCTA) has significantly expanded our assessment of diabetic patients without DR, as this represents a relatively new imaging technique that allows a noninvasive volumetric visualization of the retinal flow at specific depths^9–11^. OCTA may be employed as a tool to gauge DR progression and guide clinical practice, as OCTA-assessed macular ischemia was demonstrated to be predictive of DR progression in eyes without signs of retinopathy^12^. Noteworthy, OCTA findings on diabetic eyes without signs of DR are controversial as Rosen and colleagues demonstrated an increase in retinal perfusion near the foveal avascular zone (FAZ) in these eyes^13^. Therefore, considering that OCTA may be clinically relevant in diabetic patients and there are still controversies on OCTA changes in diabetic patients without DR, new technologies applied to OCTA imaging may be of value if applied to diabetic patients.

A new approach to OCTA, known as volume rendered three-dimensional (3D) OCTA^14–18^, has been shown to reliably quantify the macular perfusion in healthy and diabetic eyes^18^. Measurements of diabetic macular ischemia using volume rendered OCTA were obtained with two novel 3D OCTA metrics: (i) 3D perfusion density, and (ii) 3D vascular volume^18^. Three-dimensional measurements of diabetic macular ischemia gave excellent intra-session repeatability^19^. The 3D approach was designed to partially overcome limitations of conventional *en face* two-dimensional (2D) visualization, particularly in eyes with exudative macular diseases, that include segmentation artifacts (due to exudation), underestimation of flow as overlapping vessels segmented as part of the same slab may erroneously be merged, or overestimation of flow as retinal vessels crossing distinct slabs may be erroneously visualized twice on two different OCTA images (e.g. superficial capillary plexus and deep vascular complex, SCP and DVC, respectively)^9–11,16,20^.

The current study was designed to investigate 3D OCTA metrics in type 1 diabetic patients without diabetic retinopathy. Furthermore, a second purpose was to weigh the contribution of different plexuses (SCP vs DVC) to the volume rendered macular ischemia by employing a 2D visualization. We also examined correlations between clinical factors (i.e. diabetes duration and HbA1c level) and 3D OCTA metrics in patients with diabetes.

## Methods

### Study participants

This was a multicenter, retrospective observational case series that adhered to the tenets of the Declaration of Helsinki and Health Insurance Portability and Accountability Act. Written informed consent was obtained from all subjects. The local institutional review boards (San Raffaele Scientific Institute Ethics Committee and Fondazione Bietti Ethics Committee) were notified about this retrospective cohort study. Based on the Italian legislation, this kind of study doesn't require the Ethics Committee approval, as it is required only a notification.

The authors in this study identified consecutive patients with type 1 diabetes and without signs of DR as determined by clinical examination between January 2019 and October 2020. Included subjects received a complete ophthalmological evaluation that included OCTA imaging. Age, duration of diabetes, and hemoglobinA1c (HbA1c) level were noted for each participant from the patient record. The absence of DR was determined by two experienced and senior graders (MP and GQ) with fundus examination and using the Diabetic Retinopathy Severity Scale. Exclusion criteria included: (i) any other maculopathy; (ii) history of any intravitreal treatment; and (iii) history or evidence of laser treatment within the macula. Furthermore, we excluded OCTA scans with a signal strength index (SSI) lower than 7, as recommended by manufacturer. One eye for each subject was included in the analysis and this was randomly selected.

### Imaging and image processing

The swept source PLEX Elite 9000 (Carl Zeiss Meditec Inc., Dublin, CA, USA) device was employed to obtain OCTA scans.

A previously validated and semi-automated algorithm was used to obtain a 3D visualization of 3 × 3-mm OCTA data^15,16,18,21^. Briefly, a volume projection removal algorithm was used to mitigate the influence of the projection artifacts on the three-dimensional visualization^15,16,18,21^. Successively, the resulting adjusted OCTA volume scans were exported as uncompressed 8-bit raw-data file and opened in ImageJ software version 1.50 (National Institutes of Health, Bethesda, MD; available at http://rsb.info.nih.gov/ij/index.html)^22^. Subsequently, a multi-step algorithm previously outlined in details was employed^18^. In brief, the OCTA scan was re-oriented in space and a rescaling number was applied to the obtained stack using the following formula:

1 $$Z \,scale = \frac{Z \,geometric \times Z \,resolution}{XY \,resolution \times XY \,scan \,dimension }= \frac{3\times300}{1536\times3}=0.195$$

**Figure 1:**
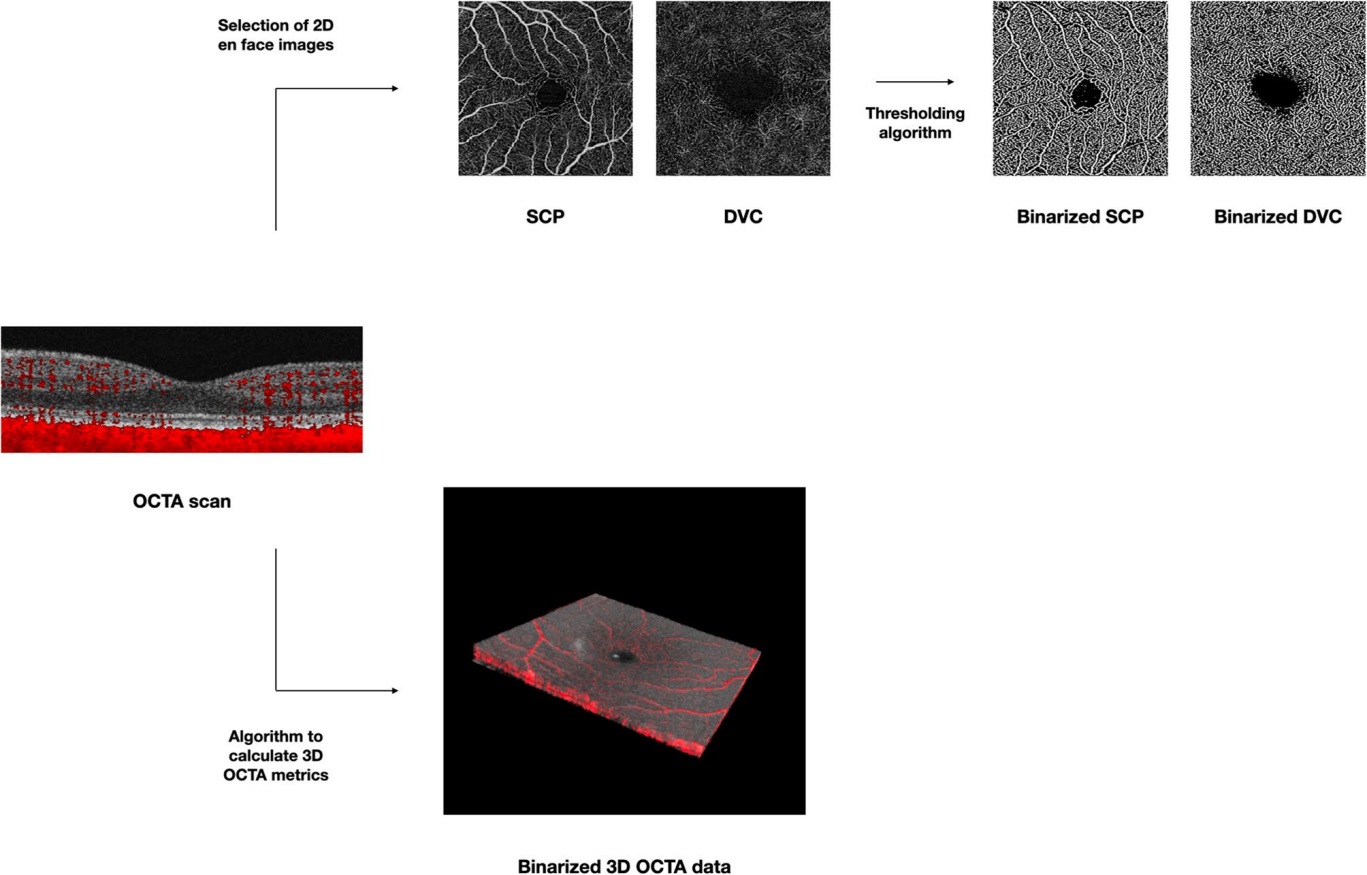
Representation of the algorithm used to process the OCTA data. For each patient, the OCTA volume scan was imported in ImageJ (https://imagej.nih.gov/ij/) and processed. The images of the superficial capillary plexus (SCP) and deep vascular complex (DVC) were binarized in order to test the SCP and DCP 2D metrics. 3D OCTA data were also processed with a thresholding algorithm. The latter process was aimed at including all those voxels falling above this threshold—displayed in red in this figure—within the neuroretina.

Successively, the “Scale” and “Make Substack” functions were executed to modify the “Z scale” and to only comprise retinal vessels, respectively. Lastly, we executed the global default threshold on the image stack (considering the stack’s histogram) and this process resulted in excluding all those voxels falling below this threshold (Fig. 1, Supplementary Videos 1 and 2). Then, we calculated the following 3D OCTA metrics: (i) 3D perfusion density and (ii) 3D vascular volume. The “make substack” function was employed to include only slabs incorporating voxels from retinal vessels (manual step in the semi-automated algorithm). While the “3D vascular volume” was determined by the whole number of voxels with a brightness superior to that of the applied threshold, the 3D perfusion density was calculated using the following formula:

2 $$3D\,perfusion\,density = \frac{3D\,vascular\,volume}{volume\,of\,neuroretina}$$

The neuroretinal volume was obtained using the “ETDRS Retina Thickness v0.1” algorithm provided by Carl Zeiss Meditec Inc and available on the Advanced Retina Imaging (ARI) portal. The latter algorithm was already employed in diabetic eyes to measure to calculate total retinal thickness and volume values for each ETDRS standard areas^13^. Two-dimensional *en face* OCTA metrics were also obtained using segmentations at the instrument’s default setting^23,24^ and applying a semi-automated threshold that has been previously used and validated in diabetic patients^25^.

### Statistical analysis

To detect departures from normality distribution, a Shapiro–Wilk’s test was performed for all variables. Means and standard deviation (SD) were computed for all quantitative variables. Independent samples Student’s t-test was used to compare quantitative variables. Pearson’s correlation was performed to evaluate the linear correlation among variables in patients with diabetes.

Statistical Package for Social Sciences (version 20.0, SPSS Inc., Chicago, IL, USA) was used to perform statistical calculations. The chosen level of statistical significance was p < 0.05.

## Results

Of the 70 subjects (70 eyes) included in this analysis, 35 (21 females) had type 1 diabetes without signs of DR and 35 (19 females) were healthy controls. Mean ± SD age was 27.2 ± 10.2 years [range 19–64 years] in the diabetic group and 31.0 ± 11.4 years [range 19–61 years] in the control group (p = 0.145). Patients and controls were all phakic. The BCVA was 0.0 ± 0.1 LogMAR in the diabetic group and 0.0 ± 0.0 LogMAR in healthy eyes (p = 0.871). Mean ± SD HbA1c level was 7.5 ± 0.7% [range 6.0–8.6%]. Mean ± SD duration of diabetes was 13.2 ± 6.0 years [range 5.0–35.0 years].

The structural OCT assessment revealed that the average macular thickness (in the tested ROI) was 321.5 ± 14.9 µm in diabetic patients and 319.6 ± 11.1 µm in controls (p = 0.562). The average macular volume was also similar in the two groups (2.89 ± 0.13 mm^3^ in diabetic subjects vs 2.87 ± 0.10 mm^3^ in healthy individuals, p = 0.455) (Table 1).

**Table 1:** Structural OCT and OCTA metrics in diabetic patients and healthy controls.

	Diabetic patients	Controls	p value
**3D OCTA metrics**			
Vascular Volume (mm^3^)	0.27 ± 0.05	0.29 ± 0.04	0.020
Perfusion density (%)	9.3 ± 1.6	10.3 ± 1.6	0.005
**2D OCTA metrics**			
SCP perfusion density (%)	34.4 ± 3.1	34.3 ± 3.8	0.899
DVC perfusion density (%)	38.9 ± 3.7	41.0 ± 3.1	0.001
**Structural OCT metrics**			
Average macular thickness (µm)	321.5 ± 14.9	319.6 ± 12.1	0.562
Average macular volume (mm^3^)	2.89 ± 0.13	2.87 ± 0.10	0.455

Data are expressed as mean±SD (standard deviation). *2D* two-dimensiona, *3D* three-dimensiona, *OCTA* optical coherence tomography angiography, *SCP* superficial capillary plexus, *DVC* deep vascular complex.

### 3D and 2D OCTA metrics

The 3D vascular volume was 0.27 ± 0.05 mm^3^ in the diabetic group and 0.29 ± 0.04 mm^3^ in the control group (p = 0.020). The 3D perfusion density was 9.3 ± 1.6% and 10.3 ± 1.6% in diabetic patients and controls, respectively (p = 0.005) (Table 1).

Using a 2D visualization, the perfusion density was lower in diabetic patients, but only at the DVC level (38.9 ± 3.7% in diabetes and 41.0 ± 3.1% in controls, p = 0.001), while no differences were detected at the SCP level (34.4 ± 3.1% and 34.3 ± 3.8% in the diabetic and healthy subjects, respectively, p = 0.899).

### Correlation analysis

In the Pearson correlation analysis (Table 2), neither 3D or 2D OCTA metrics were significantly correlated with diabetes duration and HbA1c level (Table 2).

**Table 2:** Correlation analysis between OCTA parameters and other variables (i.e. HbA1c level and diabetes duration).

	Pearson coefficient	p value
**HbA1c level**		
2D SCP perfusion density (%)	0.023	0.896
2D DVC perfusion density (%)	− 0.082	0.638
3D vascular volume (mm^3^)	0.139	0.427
3D perfusion density (%)	0.173	0.322
**Diabetes duration**		
2D SCP perfusion density (%)	− 0.239	0.167
2D DVC perfusion density (%)	0.357	0.070
3D vascular volume (mm^3^)	0.031	0.861
3D perfusion density (%)	− 0.024	0.889

*HbA1c* hemoglobin A1c, *OCTA* optical coherence tomography angiography, *2D* two-dimensional, *3D* three-dimensional, *SCP* superficial capillary plexus, *DVC* deep vascular complex.

## Discussion

In this study, we employed 3D OCTA metrics to quantitatively investigate the macular ischemia in diabetic patients without evidence of diabetic retinopathy. Overall, we observed that these eyes are characterized by a reduced volume rendered macular perfusion.

In contrast with 2D OCTA, flattening of flow data is not needed in a volume rendered 3D visualization and this demonstrated to result in an enhanced valuation of the retinal vessels^14–17^. Our group has recently assessed eyes with DR using a three-dimensional analysis^16,18,21^. In details, diabetic microaneurysms were anatomically described in vivo using a 3D visualization and this was demonstrated to be a valuable methodology for a more detailed description of these vascular alterations^16^. More importantly, we recently introduced two novel 3D OCTA metrics (3D perfusion density and 3D vascular volume)^18^. In this previous paper, we analyzed 15 diabetic and 15 healthy individuals. OCTA data were elaborated in order to obtain and compare 2D and 3D OCTA measures. This study showed a positive association between 2 and 3D parameters. Moreover, diabetic patients and healthy controls had significant differences in terms of 3D quantitative metrics. 3D OCTA metrics were also characterized by better values of intra-session repeatability, as segmentation artifacts may affect a 2D OCTA assessment, with special regard to diseased eyes^21^. In details, we analyzed 20 DR patients with macular edema who had two consecutive OCTA imaging scans obtained during the same visit. In the latter study, the intraclass correlation coefficient (ICC) ranged from 0.935 to 0.967 for 3D OCTA metrics and from 0.591 to 0.824 for 2D OCTA metrics^21^. Similarly, the coefficient of variation (CV) ranged from 1.9 to 2.0 for 3D OCTA metrics and from 2.2 to 4.2 for 2D OCTA metrics. Therefore, we concluded that the 3D OCTA-based quantifications had the highest inter-scan intra-session agreements^21^. More importantly, given that differences in inter-scan 2D OCTA metrics’ values were associated with average macular volume, we concluded that 2D OCTA metrics have lower intra-session agreements because of the high rate of segmentation errors^21^.

Data from a number of OCTA studies using a 2D approach indicate that diabetic patients without signs of diabetic retinopathy are characterized by an early macular ischemia^3–8,26^. Recently, Ashraf et al.^5^ employed 2D OCTA analysis to correlate macular perfusion with DR staging in diabetic patients. In detail, they retrospectively analyzed 396 eyes (237 diabetic patients) that were affected by different stages of DR, and observed that the macular perfusion in eyes with no to early DR was primarily reduced in the deeper vascular layers, while the SCP was mainly spared in these stages. Similarly, Carnevali et al.^4^ employed a 2D OCTA analysis on 25 patients (25 eyes) with type 1 diabetes and without DR. In agreement with the Ashraf and colleagues’ study^5^, a significant reduction in macular perfusion was detectable only at the DVC level. Accordingly, the present study demonstrated a significant reduction in DVC perfusion, while no differences were detected at the superficial level. Early SCP vascular changes may be masked by an associated increase in vascular flow and vascular dilation in this vascular layer secondary to a possible “steal phenomena” from deeper vascular layers, as previously suggested^27,28^.

We add to the literature by reporting both 3D perfusion density and vascular volume in a population of 35 type 1 diabetic patients without signs of DR compared to 35 controls in a retrospective cross-sectional study. In our study cohort of diabetic patients, both the 3D vascular volume and perfusion density were significantly reduced. As stated above, a 3D visualization was demonstrated to result in an enhanced assessment of the retinal vessels^14–17^. Moreover, the 3D OCTA-based quantification of the macular perfusion is characterized by higher levels of repeatability, in comparison with a 2D OCTA-based assessment, thus allowing a more reliable assessment of the macular ischemia^21^. Therefore, this study employing a novel and reliable technology further confirms that macular ischemia occurs early in diabetic patients. Furthermore, the estimation and comparison of either metric (i.e. 3D perfusion density and vascular volume) may allow a better comprehension of the disease processes occurring in diabetes as some disease stages may affect vascular volume more than perfusion-structure ratio and vice versa. Noteworthy, we did not find any difference in terms of neuroretinal thickness and volume values between diabetic and healthy subjects. With a conserved macular structure, a reduced vascular volume may result in a more severe ischemia. Therefore, the 3D perfusion density, which accounts for the neuroretinal volume, may constitute a more suitable metric to quantify the actual metabolic supply. This is an additional potential advantage of volume-rendered 3D in comparison with a 2D OCTA visualization, whose values are instead obtained by summing or projecting flow data within any slab and are therefore independent on the neuroretinal thickness. Future studies with longitudinal follow-up will clarify whether the 3D perfusion density may represent a predictive metric in patients with diabetes.

As noted above, the deeper retinal vessels appear to be preferentially affected in diabetic patients with no signs of DR, while the superficial vascular layer seems to be damaged later in presence of DR. This is in agreement with our results, which therefore may suggest that either a 2D OCTA assessment of the DVC perfusion or a 3D OCTA quantification of the macular perfusion may be used to detect macular ischemia in diabetic patients without DR. Assuming that the 3D OCTA metrics were demonstrated to have the highest receiver operating characteristic (ROC) curves in differentiating DR eyes from healthy eyes^18^, our results may suggest that assessing quantitative perfusion using 3D analysis is reliable and promising in patients with DR. Finally, by comparing 3D and 2D visualizations we were able to demonstrate the significant contribution of DVC to the reduced volume rendered macular perfusion. In agreement with a previous important study employing 2D OCTA^5^, we did not find any association between HbA1c levels and macular perfusion in our study cohort. Although a poor glycemic control is thought to be a relevant factor associated with vascular damage in diabetic patients, HbA1c levels may not actually reflect the historical glycemic control in these patients, but rather contemplate the control obtained in the last months. Conversely, we did not find associations between 3D OCTA metrics and duration of diabetes, which is in disagreement with previous findings obtained with 2D OCTA^5^. We may speculate that this is related to the fact that our patients are characterized by a tight range of diabetes duration (13.2 ± 6.0 years, range 5.0–35.0 years).

The present study has limitations that should be considered in the interpretation of our results. The main limitation is that projection artifacts may influence a 3D analysis. However, in order to alleviate this limitation, we employed a previously validated volume projection artifact removal algorithm^15,16^. Furthermore, a volume-rendered 3D visualization currently needs a semi-automated approach and manual adjustments and expertise are needed to properly complete this methodology. The latter aspects may limit its use in clinical practice. Future advancements in this methodology with automated algorithms may result in broader application. Moreover, this is a cross-sectional study without data on follow-up. Future longitudinal studies may clarify whether 3D OCTA metrics may have a prognostic role in diabetic patients.

In conclusion, the present study used a semi-automated algorithm to obtain a 3D viewing of the retinal macular perfusion in type 1 diabetic patients with no diabetic retinopathy. In our study, patients with diabetes and no DR have a reduced volume rendered macular perfusion compared to control healthy eyes. Importantly, we demonstrated the role of DVC to the reduced volume rendered macular perfusion. If replicated in future studies, 3D OCTA metrics may prove to be useful parameters for evaluating diabetic patients. Volume rendered OCTA may become a novel tool for monitoring the efficacy of novel therapeutic approaches to prevent the development of diabetic retinopathy.

## Supplementary Information

Supplementary Video 1.

Supplementary Video 2.

Supplementary Information 1.

## Data Availability

The data used to support the findings of this study are available from the corresponding author upon request.

## References

[1] Sim DA, Keane PA, Zarranz-Ventura J (2013). Predictive factors for the progression of diabetic macular ischemia. Am. J. Ophthalmol..

[2] Beltramo E, Porta M (2013). Pericyte loss in diabetic retinopathy: Mechanisms and consequences. Curr. Med. Chem..

[3] Dimitrova G, Chihara E, Takahashi H, Amano H, Okazaki K (2017). Quantitative retinal optical coherence tomography angiography in patients with diabetes without diabetic retinopathy. Investig. Ophthalmol. Vis. Sci..

[4] Carnevali A, Sacconi R, Corbelli E (2017). Optical coherence tomography angiography analysis of retinal vascular plexuses and choriocapillaris in patients with type 1 diabetes without diabetic retinopathy. Acta Diabetol..

[5] Ashraf M, Sampani K, Clermont A (2020). Vascular density of deep, intermediate and superficial vascular plexuses are differentially affected by diabetic retinopathy severity. Investig. Ophthalmol. Vis. Sci..

[6] Simonett JM, Scarinci F, Picconi F (2017). Early microvascular retinal changes in optical coherence tomography angiography in patients with type 1 diabetes mellitus. Acta Ophthalmol..

[7] Choi W, Waheed NK, Moult EM (2017). Ultrahigh speed swept source optical coherence tomography angiography of retinal and choriocapillaris alterations in diabetic patients with and without retinopathy. Retina.

[8] De Carlo TE, Chin AT, Bonini Filho MA (2015). Detection of microvascular changes in eyes of patients with diabetes but not clinical diabetic retinopathy using optical coherence tomography angiography. Retina.

[9] Borrelli E, Sadda SR, Uji A, Querques G (2019). Pearls and pitfalls of optical coherence tomography angiography imaging: A review. Ophthalmol. Ther..

[10] Borrelli E, Sarraf D, Freund KB, Sadda SR (2018). OCT angiography and evaluation of the choroid and choroidal vascular disorders. Prog. Retina Eye Res..

[11] Spaide RRF, Fujimoto JG, Waheed NK, Sadda SR, Staurenghi G (2017). Optical coherence tomography angiography. Prog. Retina Eye Res..

[12] Sun Z, Tang F, Wong R (2019). OCT angiography metrics predict progression of diabetic retinopathy and development of diabetic macular edema: A prospective study. Ophthalmology.

[13] Santos T, Warren LH, Santos AR (2020). Swept-source OCTA quantification of capillary closure predicts ETDRS severity staging of NPDR. Br. J. Ophthalmol..

[14] Spaide RF, Suzuki M, Yannuzzi LA, Matet A, Behar-Cohen F (2017). Volume-rendered angiographic and structural optical coherence tomography angiography of macular telangiectasia type 2. Retina.

[15] Borrelli E, Sacconi R, Klose G, de Sisternes L, Bandello F, Querques G (2019). Rotational Three-dimensional OCTA: A notable new imaging tool to characterize type 3 macular neovascularization. Sci. Rep..

[16] Borrelli E, Sacconi R, Brambati M, Bandello F, Querques G (2019). In vivo rotational three-dimensional OCTA analysis of microaneurysms in the human diabetic retina. Sci. Rep..

[17] Zhang J, Qiao Y, Sarabi MS (2019). 3D shape modeling and analysis of retinal microvasculature in OCT-angiography images. IEEE Trans. Med. Imaging.

[18] Borrelli E, Sacconi R, Querques L, Battista M, Bandello F, Querques G (2020). Quantification of diabetic macular ischemia using novel three-dimensional optical coherence tomography angiography metrics. J. Biophotonics..

[19] Borrelli E, Parravano M, Costanzo E (2020). Using three-dimensional OCTA metrics improves repeatability on quantification of ischemia in eyes with diabetic macular edema. Retina.

[20] Ghasemi Falavarjani K, Habibi A, Anvari P (2020). Effect of segmentation error correction on optical coherence tomography angiography measurements in healthy subjects and diabetic macular oedema. Br. J. Ophthalmol..

[21] Borrelli E, Parravano M, Costanzo E (2020). Using three-dimensional OCTA metrics improves repeatability on quantification of ischemia in eyes with diabetic macular edema. Retina.

[22] Schneider CA, Rasband WS, Eliceiri KW (2012). NIH image to ImageJ: 25 Years of image analysis. Nat. Methods.

[23] Parravano M, Costanzo E, Borrelli E (2020). Appearance of cysts and capillary non perfusion areas in diabetic macular edema using two different OCTA devices. Sci. Rep..

[24] Rabiolo A, Gelormini F, Marchese A (2018). Macular perfusion parameters in different angiocube sizes: Does the size matter in quantitative optical coherence tomography angiography?. Investig. Ophthalmol. Vis. Sci..

[25] Hirano T, Kitahara J, Toriyama Y, Kasamatsu H, Murata T, Sadda S (2019). Quantifying vascular density and morphology using different swept-source optical coherence tomography angiographic scan patterns in diabetic retinopathy. Br. J. Ophthalmol..

[26] Scarinci F, Picconi F, Giorno P (2018). Deep capillary plexus impairment in patients with type 1 diabetes mellitus with no signs of diabetic retinopathy revealed using optical coherence tomography angiography. Acta Ophthalmol..

[27] Onishi AC, Nesper PL, Roberts PK (2018). Importance of considering the middle capillary plexus on OCT angiography in diabetic retinopathy. Investig. Ophthalmol. Vis. Sci..

[28] Zhang M, Hwang TS, Dongye C, Wilson DJ, Huang D, Jia Y (2016). Automated quantification of nonperfusion in three retinal plexuses using projection-resolved optical coherence tomography angiography in diabetic retinopathy. Invest. Ophthalmol. Vis. Sci..

