# Supplementary material for: Volume rendered 3D OCTA assessment of macular ischemia in patients with type 1 diabetes and without diabetic retinopathy
Source: Sci Rep. 2021 Oct 5;11:19793. doi: 10.1038/s41598-021-99297-7 (PMC8492730; doi:10.1038/s41598-021-99297-7)
Supplement: Supplementary file 3 — Supplementary Information 1. [file 41598_2021_99297_MOESM3_ESM.docx]

**Video 1. Rotational 3D visualization of the macula from a healthy subject.**

The voxels from retinal vessels above the thresholding algorithm are visualized in red.

**Video 2. Rotational 3D visualization of the macula from a patient with diabetes and no diabetic retinopathy.**

The voxels from retinal vessels above the thresholding algorithm are visualized in red.
